# Triptolide alleviates rheumatoid arthritis via modulating gut microbiota – intestinal barrier – TLR4/NF-κB axis

**DOI:** 10.1515/biol-2025-1290

**Published:** 2026-06-08

**Authors:** Xuekang Pan, Zeyuan Jin, Jiaqi Bi, Huigen Lu, Gang Chen

**Affiliations:** Department of Orthopaedics, The Second Affiliated Hospital of Jiaxing University, Jiaxing, 314000, China

**Keywords:** triptolide, rheumatoid arthritis, intestinal bacteria, TLR4/NF-κB

## Abstract

Rheumatoid arthritis (RA) is a chronic autoimmune disease driven by dysregulated inflammation and intestinal microbiota-gut-joint axis dysfunction. While triptolide, a natural immunosuppressive compound, exhibits anti-RA activity, its mechanism linking gut microbiota modulation, intestinal barrier repair, and inflammatory signaling remains unclear. This study aimed to investigate whether triptolide alleviates adjuvant-induced arthritis (AIA) in mice by regulating the gut microbiota-intestinal barrier-toll-like receptor 4 (TLR4)/nuclear factor-κB (NF-κB) axis. The anti-inflammatory effect of triptolide was assessed using lipopolysaccharide (LPS)-induced RAW 264.7 cells and primary peritoneal macrophages. The anti-RA efficacy was investigated in AIA mice through detecting inflammatory factors, intestinal barrier integrity and gut microbiota. The results showed that triptolide decreased inflammatory factors in both RAW 264.7 cells and primary peritoneal macrophages. Meanwhile, triptolide dose-dependently reduced AI scores and paw swelling in AIA mice (*p* < 0.05 vs. AIA group), along with restored intestinal barrier integrity by upregulating ZO-1 and Occludin (*p* < 0.05 vs. AIA group) and modulated gut microbiota. Mechanistically, triptolide inhibited TLR4/NF-κB activation, reducing downstream pro-inflammatory cytokines (interleukin-6 (IL-6), tumor necrosis factor *α* (TNF-α)). In sum, these findings reveal that triptolide alleviates AIA by modulating the gut microbiota, repairing the intestinal barrier, and suppressing TLR4/NF-κB signaling. This highlights a novel “microbiota-gut-joint” regulatory mechanism of triptolide, supporting its potential as a therapeutic agent targeting RA’s multifactorial pathogenesis.

## 1 Introduction

Rheumatoid arthritis (RA) is a chronic, systemic autoimmune disease characterized by synovial inflammation, cartilage degradation, and joint destruction, affecting nearly 1 % of the global population with a rising incidence [1]. The disease disproportionately impacts women aged 40–60 years, imposing substantial clinical and socioeconomic burdens due to persistent pain, disability, and increased risk of comorbidities [1]. Despite advances in disease-modifying antirheumatic drugs (DMARDs) and biologics, up to 40 % of patients exhibit incomplete response or develop treatment resistance, highlighting the need for novel therapeutic strategies targeting understudied pathogenic pathways [2].

Emerging evidence identifies the gut microbiota as a critical regulator of RA pathogenesis, with the “gut-joint axis” gaining recognition as a key driver of autoimmune activation [3]. Genome-wide association Study (GWAS) has linked RA susceptibility to genes involved in microbial sensing (e.g., toll-like receptor 4 (TLR4), nucleotide-binding oligomerization domain 2 (NOD2)) [4], while clinical studies report distinct gut microbial signatures in RA patients compared to healthy controls [5]. For example, Andrew et al. observed that *Bacillus* and *Lactobacillus*, two potentially pathogenic bacteria, are characteristic of RA, with their relative abundance being two to three times higher in RA patients compared with healthy individuals [6]. Mechanistically, gut dysbiosis in RA disrupts intestinal barrier integrity, permitting translocation of microbial antigens and metabolites into systemic circulation [7]. Chen et al. showed that Collinsella aerofaciens – enriched in RA patients – downregulates tight junction proteins (e.g., zonula occludens-1 [ZO-1]) and increases gut permeability, triggering mucosal immune activation [8]. This “leaky gut” phenotype promotes the expansion of pro-inflammatory T helper 17 (Th17) cells and the production of autoantibodies (e.g., anti-citrullinated protein antibodies [ACPAs]), which cross-react with joint tissues to initiate synovitis [9]. Among the signaling pathways connecting gut microbiota to systemic inflammation, the TLR4/nuclear factor-κB (NF-κB) axis emerges as a critical driver of RA pathogenesis. TLR4, a pattern recognition receptor (PRR) expressed on immune cells (macrophages, dendritic cells) and intestinal epithelial cells, recognizes microbial-associated molecular patterns (MAMPs) such as lipopolysaccharide (LPS) - a major component of Gram-negative bacteria enriched in RA gut dysbiosis. In RA patients, elevated serum LPS levels correlate with disease activity, and TLR4 expression is upregulated in synovial tissue, where it promotes pro-inflammatory cytokine secretion (TNF-α, IL-6, IL-1β) via NF-κB activation. Collectively, these findings position the gut microbiota as a tractable target for RA intervention, yet few therapies explicitly modulate this axis.

Traditional Chinese medicine offers alternative approaches to RA management, with *Tripterygium wilfordii* Hook F (TwHF) demonstrating efficacy in randomized controlled trials [10]. A phase III trial showed TwHF extract was non-inferior to methotrexate in achieving ACR20 responses, with comparable safety profiles. Triptolide, the principal active compound, modulates immune responses by suppressing the production of pro-inflammatory cytokines and reducing the activity of immune cells involved in RA pathogenesis [11]. Its pharmacological actions include inhibiting the migration of B cells and monocytes and decreasing pro-inflammatory cytokine secretion [12]. Additionally, triptolide alleviates oxidative stress by enhancing antioxidant enzyme activity and reducing reactive oxygen species (ROS) generation [13]. These combined effects highlight the potential of triptolide as a promising therapeutic agent for RA management. However, critical gaps remain: (1) Does triptolide modulate the gut microbiota in RA? (2) If so, does this modulation contribute to intestinal barrier repair and immune suppression in RA? (3) What are the downstream molecular pathways linking triptolide-microbiota interactions to reduced joint inflammation? Addressing these questions could unravel a novel mechanism for triptolide’s action and validate the gut microbiota as a therapeutic target in RA.

Given the established role of gut dysbiosis in RA and triptolide’s emerging microbiota-modulatory properties, we hypothesized that triptolide alleviates RA by restoring gut microbial balance, thereby reinforcing intestinal barrier integrity and suppressing systemic inflammation. To test this, we analyzed gut microbial composition, intestinal barrier function, and inflammatory signaling in a murine adjuvant-induced arthritis (AIA) model treated with triptolide. The objectives were to: (1) characterize triptolide-induced changes in gut microbial diversity and key taxa; (2) evaluate its impact on intestinal barrier proteins (ZO-1, occludin) and pro-inflammatory cytokines; and (3) explore associations between microbial shifts and therapeutic outcomes. By integrating pro-inflammatory cytokines detection, intestinal barrier function evaluation, and gut microbiota analyses, we aim to clarify the microbiota-mediated mechanisms underlying triptolid’s anti-RA effects, providing a rationale for its clinical application as a gut microbiota-targeted therapy.

## 2 Materials and methods

### 2.1 Animals, models, and administration

Male BALB/c mice (10–12 weeks old, approximately 20 g) were obtained from Canvens Experimental Animal Co., Ltd. (Changzhou, China). All experimental procedures followed the ethical guidelines approved by the Jiaxing Second Hospital Ethics Committee (JUMC2023-007). Mice were housed under specific pathogen-free (SPF) conditions with free access to water and a standard chow diet. Mice were randomly divided into six groups (*n* = 6 per group): Control, adjuvant-induced arthritis (AIA), high-dose triptolide (Htrip, 0.5 mg kg^−1^), medium-dose triptolide (Mtrip, 0.25 mg kg^−1^), low-dose triptolide (Ltrip, 0.125 mg kg^−1^), and celecoxib (Cele).

To induce an RA mouse model, Complete Freund’s adjuvant (CFA) was selected due to its well-established ability to closely replicate the pathological features of RA. Although the collagen-induced arthritis (CIA) model is widely used, the adjuvant-induced arthritis (AIA) model was chosen for several reasons. First, it ensures consistency with previous research, enabling more reliable comparison and data interpretation. Second, the CFA-induced AIA model provides high reproducibility and accurately reflects the inflammatory and immune responses characteristic of RA. Third, compared with the CIA model, AIA is simpler to perform and yields more uniform induction across animals. Except for Control group, other groups were intraperitoneally injected with 50 μL CFA (1 mg/mL) into the ankle cavity to establish the AIA model [14], and control group was intraperitoneally injected with 50 μL physiological saline. Simultaneously, control and AIA groups were given saline by physiological saline, while Ltrip, Mtrip and Htrip groups were given corresponding dosage of triptolide via intraperitoneal injection. The dosage of triptolide was carefully selected to give an optimal balance of efficacy and toxicity based on literature reports [15], [16], [17]. Cele group was given celecoxib (10 mg/kg) by gavage, served as a reference treatment for comparison. During experiment, disease severity and progression are evaluated by Arthritis Index (AI) scoring, body weight measurement, paw thickness assessment, and histopathological analysis. After four weeks of treatment, blood, colon, and synovial tissue samples were collected. Peritoneal macrophages were isolated from each group using 5 mL phosphate-buffered saline (PBS). All animal procedures followed the institutional guidelines for the care and use of laboratory animals.

**Ethical approval:** The research related to animal use has been complied with all the relevant national regulations and institutional policies for the care and use of animals, and has been approved by the Jiaxing Second Hospital Ethics Committee (JUMC2023-007).

### 2.2 Cell culture

RAW 264.7 cells were obtained from the American Type Culture Collection (Manassas, USA), and primary peritoneal macrophages were isolated from mice through peritoneal lavage. Following established protocols, both RAW 264.7 cells and primary macrophages were cultured in high-glucose DMEM at 37 °C in a humidified incubator containing 5 % CO_2_ [18]. Cells were stimulated with lipopolysaccharide (LPS, 1 μg/mL) and subsequently treated with varying concentrations of triptolide [high (Htrip, 1 μM), medium (Mtrip, 0.5 μM), or low (Ltrip, 0.1 μM)] or with celecoxib for downstream analyses.

### 2.3 Physical parameters and cytokines

Body weight, AI score, and paw swelling were measured as described previously [19]. Arthritis severity was graded on a 0–4 scale: 0, no symptoms; 1, mild erythema and swelling; 2, moderate edema; 3, severe edema and limited limb use; and 4, complete loss of limb function due to swelling. Paw thickness was measured daily using a micrometer caliper. Cytokine levels in serum from different groups were quantified using enzyme-linked immunosorbent assay (ELISA) kits.

### 2.4 Histomorphology

Ankle joints from each group were fixed in formalin for 24 h and decalcified for one month before histological analysis. Intestinal segments were fixed in 4 % paraformaldehyde, embedded, and sectioned. The sections were stained with hematoxylin and eosin (H&E), and histological images were captured under a light microscope.

### 2.5 Quantitative real-time polymerase chain reaction (qRT-PCR)

Total RNA was extracted from intestinal tissues using TRIzol reagent, and complementary DNA (cDNA) was synthesized using a commercial reverse transcription kit (Thermo Fisher Scientific, IL, USA). The primers used in the analysis were as follows: *Gapdh FP 5′-TGG​ATA​AGC​AGG​GCG​GGA-3′*, *RP 5′-CCA​ATA​CGG​CCA​AAT​CCG​TTC-3′; ZO-1 FP 5′-GAT​GTT​TAT​GCG​GAC​GGT​GG-*3′, RP 5′-CAT​TGC​TGT​GCT​CTT​AGC​GG-3′; Occludin FP 5′-AGG​TGA​ATG​GGT​CA-*CCG​AG-3′, RP 5′-AGG​CTC​CCA​AGA​TAA​GCG​AAC-3′.* Each 10 μL PCR reaction contained 5 μL of 2 × SYBR Green PCR buffer, 0.5 μL of each primer (10 μM), 1 μL of cDNA template (5 ng), and 3 μL of double-distilled water. Amplification was performed on a QuantStudio 5 Real-Time PCR System with the following program: 50 °C for 2 min, followed by 40 cycles of 95 °C for 15 s and 60 °C for 1 min, ending with a 95 °C hold. The relative mRNA expression levels were calculated using the 2^−ΔΔCt^ method, with GAPDH serving as the internal reference gene.

### 2.6 Western blot analysis

Tissue samples or cultured cells were homogenized in RIPA lysis buffer containing 1 mmol/L PMSF and lysed on ice. The lysates were centrifuged at 12,000 rpm for 10 min at 4 °C, and protein concentrations were determined using the BCA assay. Equal amounts of protein were separated by SDS-PAGE and transferred to PVDF membranes. Membranes were blocked with serum for 1 h at room temperature and incubated overnight at 4 °C with primary antibodies: anti-ZO-1 (1:2000, Proteintech), anti-Occludin (1:1000, Proteintech), anti-GAPDH (1:10000, Proteintech), anti-TLR4 (1:500, Proteintech), and anti–NF–κB (1:2000, Abcam). After washing, membranes were incubated for 2 h at room temperature with HRP-conjugated secondary antibodies (goat anti-rabbit IgG or goat anti-mouse IgG, 1:5,000, Proteintech). Protein bands were visualized using a ChemiDoc XRS + Imaging System (Bio-Rad, Carlsbad, CA, USA), and band intensities were quantified with ImageJ software. Target protein expression levels were normalized to total protein.

### 2.7 16S rRNA gene sequencing

Fecal samples were collected from mice, and total DNA was extracted using a commercial DNA extraction kit. DNA concentration and purity were assessed with a NanoDrop spectrophotometer. Amplification primers were designed according to previously reported sequences [19]. The 275–450 bp insertion fragments were sequenced using the Illumina NovaSeq platform. Raw sequencing data were processed to remove low-quality reads and generate clean data. Redundant sequences were eliminated using the Mothur software package. Operational taxonomic units (OTUs) were annotated to determine species composition and taxonomic distribution across samples. Microbial abundance and diversity were assessed using the Chao1 index, ACE index, Shannon index, and Simpson index. Beta diversity was analyzed using principal component analysis (PCA) to evaluate similarities and differences among microbial communities across groups.

### 2.8 Statistical analysis

Statistical analysis was performed using SPSS software (version 22.0). Data are presented as mean ± standard deviation (SD). To assess the normality of continuous variables (e.g., Arthritis Index [AI] scores, paw thickness, body weight changes, and cytokine levels), we employed the Shapiro-Wilk test, which is preferred for small sample sizes (*n* = 6 per group in our study) due to its higher statistical power compared to alternatives like the Kolmogorov-Smirnov test. Meanwhile, Levene’s test was used to assess variance homogeneity across groups. Since both assumptions were met, parametric tests (One-way ANOVA) were deemed appropriate for subsequent analyses. For comparisons of means across the six experimental groups (Control, AIA, Htrip, Mtrip, Ltrip, and Cele), we first performed a One-way Analysis of Variance (One-way ANOVA) to evaluate overall group differences. If the ANOVA indicated a significant main effect (*p* < 0.05), we applied Tukey’s Honestly Significant Difference (HSD) post-hoc test to conduct pairwise comparisons between all groups. A p-value of *p < 0.05 was considered statistically significant, with **p < 0.01 and ***p < 0.001 indicating higher levels of significance; “ns” denotes no statistically significant difference.

## 3 Results

### 3.1 *In vitro* study

#### 3.1.1 Triptolide ameliorates the inflammatory cytokines

**Figure 1:**
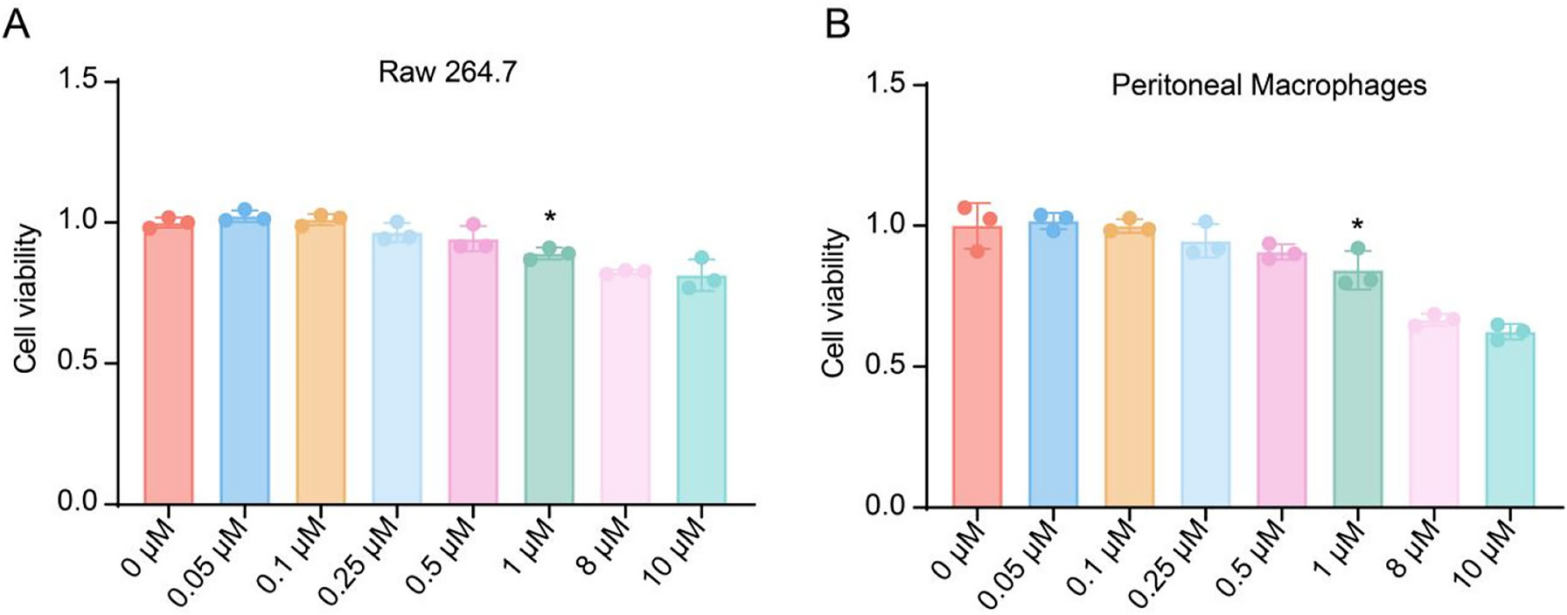
CCK8 assay. (A) Cell viability of RAW264.7 cells cultured with various concentrations of triptolide for 24 h; (B) Cell viability of peritoneal macrophages cultured with various concentrations of triptolide for 24 h. One-way Analysis of Variance (One-way ANOVA) followed to Tukey’s Honestly Significant Difference (HSD) post-hoc test to evaluate overall group differences. ^*^ *p* < 0.05 vs. control group.

Cell-Counting-Kit-8 (CCK-8) assay results indicated that triptolide at concentrations below 0.5 μM for 24 h did not significantly affect the viability of RAW264.7 cells (Figure 1A) or primary peritoneal macrophages (Figure 1B). However, cell viability declined when concentrations exceeded 1 μM. To further evaluate the anti-inflammatory potential of triptolide, LPS-induced RAW264.7 cells and primary peritoneal macrophages were treated with 0.1 μM, 0.5 μM, or 1 μM triptolide.

**Figure 2:**
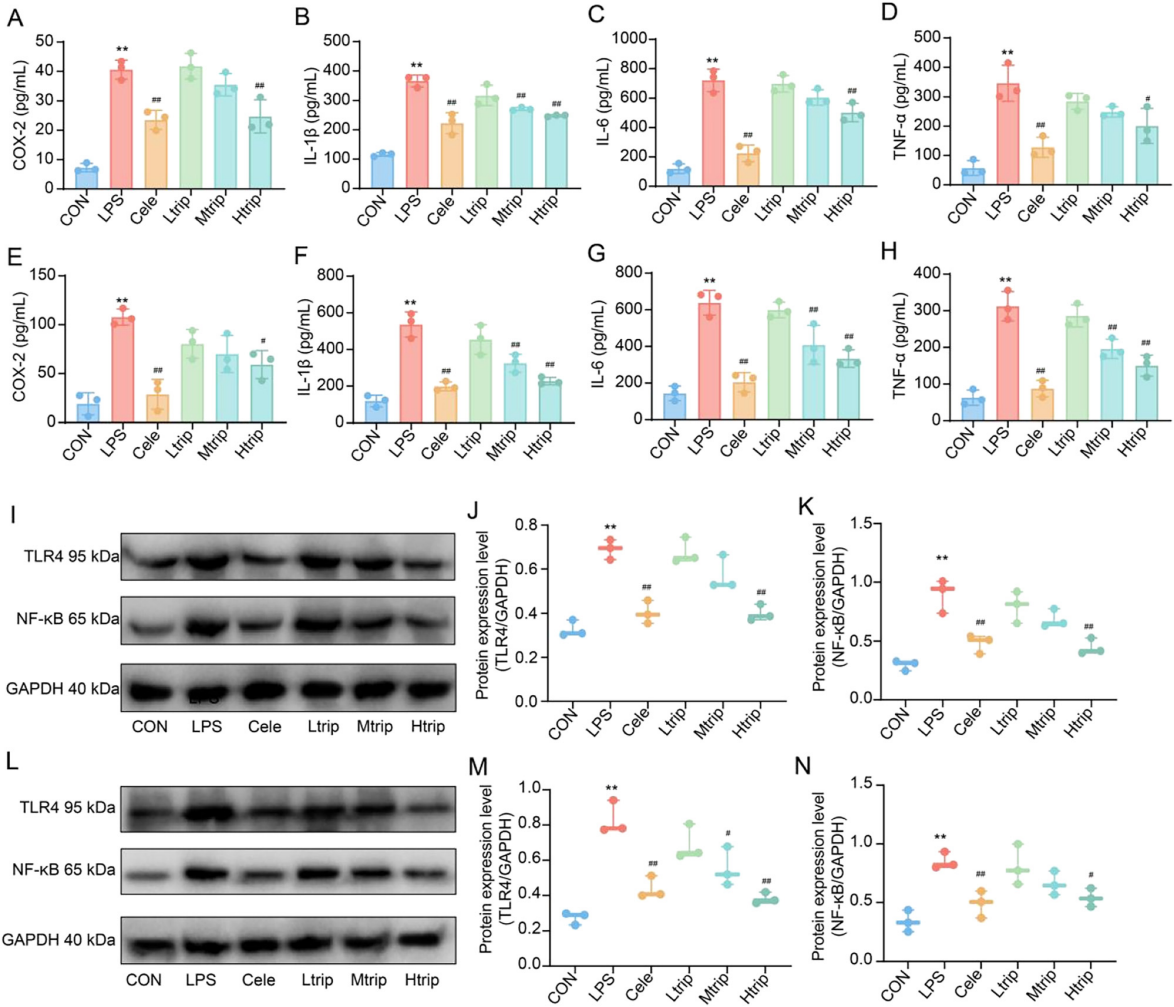
Effects of triptolide on inflammatory cytokines and the TLR4/NF-κB signaling pathway. Effects of celecoxib or triptolide on COX-2, IL-1β, IL-6 and TNF-α in LPS-induced RAW264.7 cells. The levels of COX-2 (A), IL-1β (B), IL-6 (C) and TNF-α (D) in the supernatant were determined by ELISA. Data show mean ± SD values of three independent experiments. ^**^ *p* < 0.01 vs. control group, ^#^ *p* < 0.05, ^##^ *p* < 0.01 vs. LPS group. Effects of celecoxib or triptolide on COX-2, IL-1β, IL-6 and TNF-α in peritoneal macrophages. The levels of COX-2 (E), IL-1β (F), IL-6 (G) and TNF-α (H) in the supernatant were determined by ELISA. Data show mean ± SD values of three independent experiments. ^**^ *p* < 0.01 vs. control group, ^#^ *p* < 0.05, ^##^ *p* < 0.01 vs. LPS group. Effects of triptolide on TLR4 and NF-κB in LPS-stimulated Raw 264.7 cells were measured by Western blotting analysis (I) Representative Western blot of target protein TLR4 and NF-κB expression. (J-K) Graph representing semi-quantitative analysis of the TLR4 and NF-κB proteins in each group, respectively. CON: Control group, LPS: Lipopolysaccharide group, Cele: Celecoxib group, Ltrip: low dose triptolide group, Mtrip: medium dose triptolide group, Htrip: high dose triptolide group. Data are presented as the means ± SD from three independent experiments. ^**^ *p* < 0.01 vs. control group, ^#^ *p* < 0.05, ^##^ *p* < 0.01 vs. LPS group. Effects of triptolide on TLR4 and NF-κB in peritoneal macrophages were measured by Western blotting analysis (L) Representative Western blot of target protein TLR4 and NF-κB expression. (M-N) Graph representing semi-quantitative analysis of the TLR4 and NF-κB proteins in each group, respectively. CON: Control group, LPS: Lipopolysaccharide group, Cele: Celecoxib group, Ltrip: low dose triptolide group, Mtrip: medium dose triptolide group, Htrip: high dose triptolide group. Data are presented as the means ± SD from three independent experiments. ^**^ *p* < 0.01 vs. control group, ^#^ *p* < 0.05, ^##^ *p* < 0.01 vs. LPS group. One-way Analysis of Variance (One-way ANOVA) followed to Tukey’s Honestly Significant Difference (HSD) post-hoc test to evaluate overall group differences.

Given previous findings demonstrating the anti-inflammatory effects of triptolide *in vivo*, the present study aimed to assess its protective role against LPS-induced inflammation *in vitro*. Following LPS stimulation, both cell types released pro-inflammatory cytokines. ELISA analysis revealed that triptolide significantly reduced the production of COX-2, IL-1, IL-6, and TNF-α in a dose-dependent manner (*p* < 0.05) (Figure 2A–H). These findings were consistent with the *in vivo* results, indicating that triptolide effectively mitigates cellular inflammation in LPS-stimulated macrophages.

#### 3.1.2 Triptolide on protein expression of the TLR4/NF-κB pathway

The impact of triptolide on the TLR4/NF-κB signaling pathway in RAW264.7 cells and primary peritoneal macrophages was examined using Western blot analysis. Triptolide treatment significantly reduced the protein expression of TLR4 and NF-κB in RAW264.7 cells (*p* < 0.05) (Figure 2I–K). In primary macrophages, TLR4 expression was markedly downregulated following medium- and high-dose triptolide treatment (*p* < 0.05), while NF-κB expression was significantly suppressed at the high dose (*p* < 0.05) (Figure 2L–N). The observed reduction in TLR4 and NF-κB expression corresponded with decreased levels of inflammatory cytokines after triptolide administration. Combined with the *in vivo* findings, these results suggest that triptolide may attenuate inflammation by inhibiting activation of the TLR4/NF-κB signaling pathway.

### 3.2 *In vivo* study

#### 3.2.1 Triptolide improves the weight and ameliorates the arthritic symptoms in AIA mice

**Figure 3:**
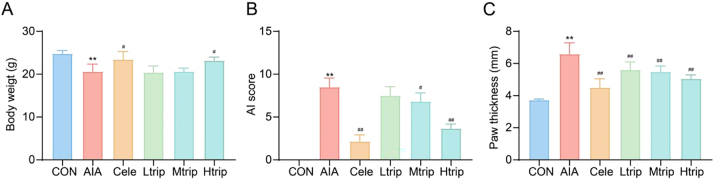
Effects of triptolide on body weight, arthritic index (AI) score, and paw thickness in AIA mice. (A) weight, (B) AI score, (C) paw thickness; AI: arthritic index; CON: Control group; AIA: adjuvant-induced arthritis group; Cele: Celecoxib group; Ltrip: low-dose triptolide group; Mtrip: medium-dose triptolide group; Htrip: high-dose triptolide group. Data are presented as the means ± SD (n=6 mice per group). ^**^ *p* < 0.01 vs. CON group, ^#^ *p* < 0.05, ^##^ *p* < 0.01 vs. AIA model group. One-way Analysis of Variance (One-way ANOVA) followed to Tukey’s Honestly Significant Difference (HSD) post-hoc test to evaluate overall group differences.

To establish the AIA model, mice received intra-articular injections of CFA, and the progression of arthritis was monitored for four weeks. AI scores, paw thickness, and body weight were recorded weekly. Histological examination of ankle joints confirmed synovial hyperplasia and inflammatory cell infiltration, verifying successful model induction. Control mice injected with saline were used to distinguish AIA-specific inflammatory responses. Following the onset of arthritis, mice exhibited a gradual decline in body weight, accompanied by significant increases in AI scores and paw swelling compared with the control group (*p* < 0.01). Oral administration of high-dose triptolide significantly prevented body weight loss and even promoted weight gain (*p* < 0.01) (Figure 3A). Treatment with triptolide at different doses reduced paw thickness in AIA mice (*p* < 0.01), while medium- and high-dose treatments markedly improved AI scores (*p* < 0.01) (Figure 3B-C). These findings indicate that triptolide effectively alleviates arthritic symptoms and mitigates disease-related weight loss in AIA mice.

#### 3.2.2 Triptolide improves pathological results of synovial tissue and intestinal tissue in AIA mice

**Figure 4:**
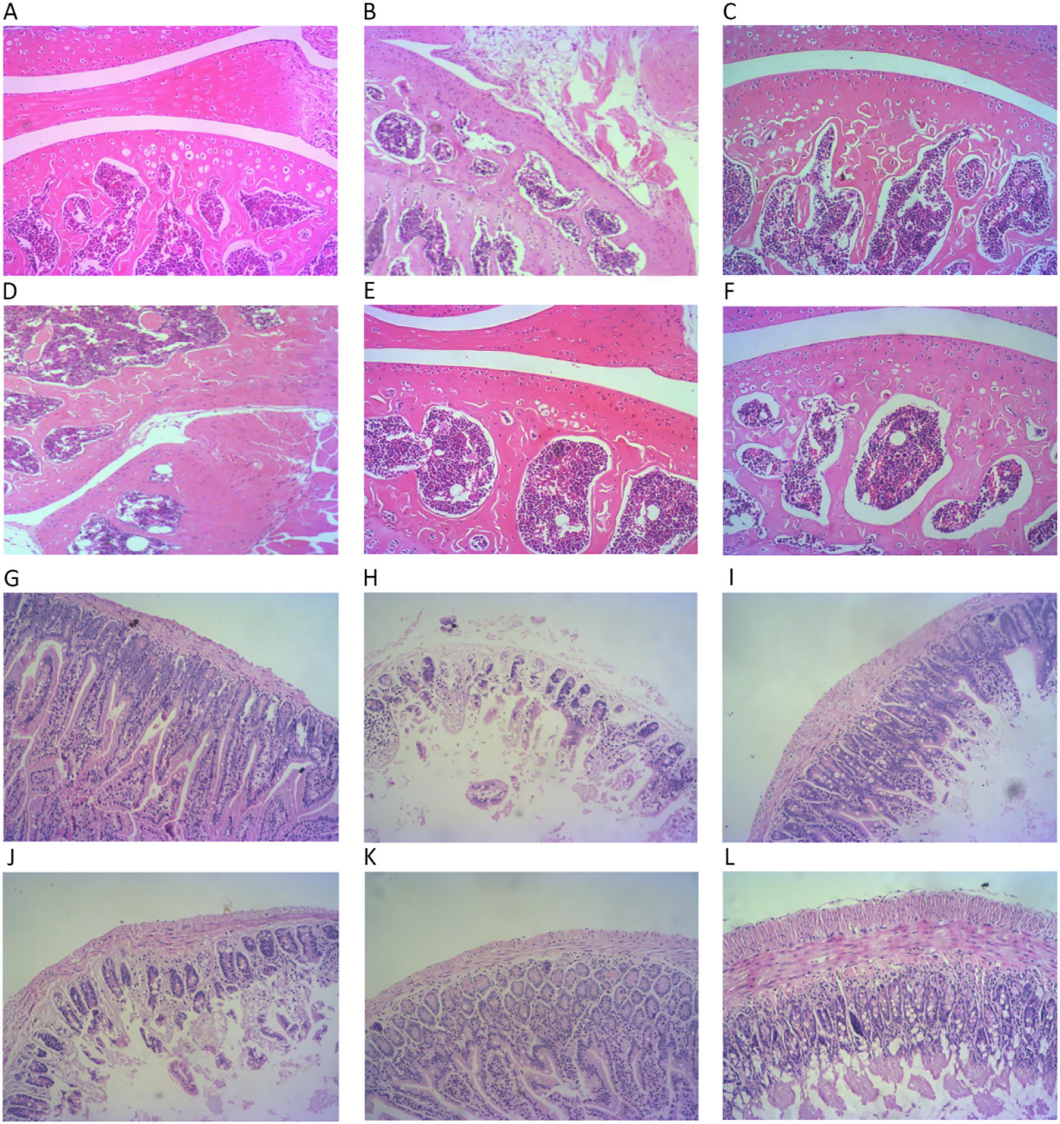
Histopathological changes in synovial and intestinal tissues of AIA mice. (A) CON group, (B) AIA group, (C) Cele group, (D) Ltrip group, (E) Mtrip group, (F) Ltrip group. CON: Control group, AIA: adjuvant-induced arthritis group, Cele: Celecoxib group, Ltrip: low-dose triptolide group, Mtrip: medium-dose triptolide group, Htrip: high-dose triptolide group. Pathological changes of intestinal tissue in the (G) CON group, (H) AIA group, (I) Cele group, (J) Ltrip group, (K) Mtrip group, (L) Ltrip group. CON: Control group, AIA: adjuvant-induced arthritis group, Cele: Celecoxib group, Ltrip: low-dose triptolide group, Mtrip: medium-dose triptolide group, Htrip: high-dose triptolide group.

H&E staining revealed pronounced inflammatory cell infiltration, extensive synovial hyperplasia, cartilage destruction, and bone erosion in the ankle joints of AIA model mice compared with controls. Treatment with medium- or high-dose triptolide markedly reduced inflammatory infiltration and synovial proliferation. Joints from the Htrip and Mtrip groups displayed only mild narrowing of the joint space, with intact cartilage and bone structures, indicating significant attenuation of synovial pathology (Figure 4A–F).

Histological examination of the ileum further supported these findings. Control mice exhibited intact villi, organized glandular architecture, and no signs of inflammation. In contrast, AIA mice showed disrupted ileal morphology characterized by shortened and damaged villi, partial detachment from the basement membrane, and marked inflammatory infiltration. Treatment with medium- or high-dose triptolide preserved intestinal integrity, maintaining most villi in normal form and substantially reducing inflammatory damage. The intestinal tissues of triptolide-treated mice displayed clear histological improvement compared with the AIA group (Figure 4G–L).

#### 3.2.3 Triptolide ameliorates the inflammatory cytokines in AIA mice

**Figure 5:**
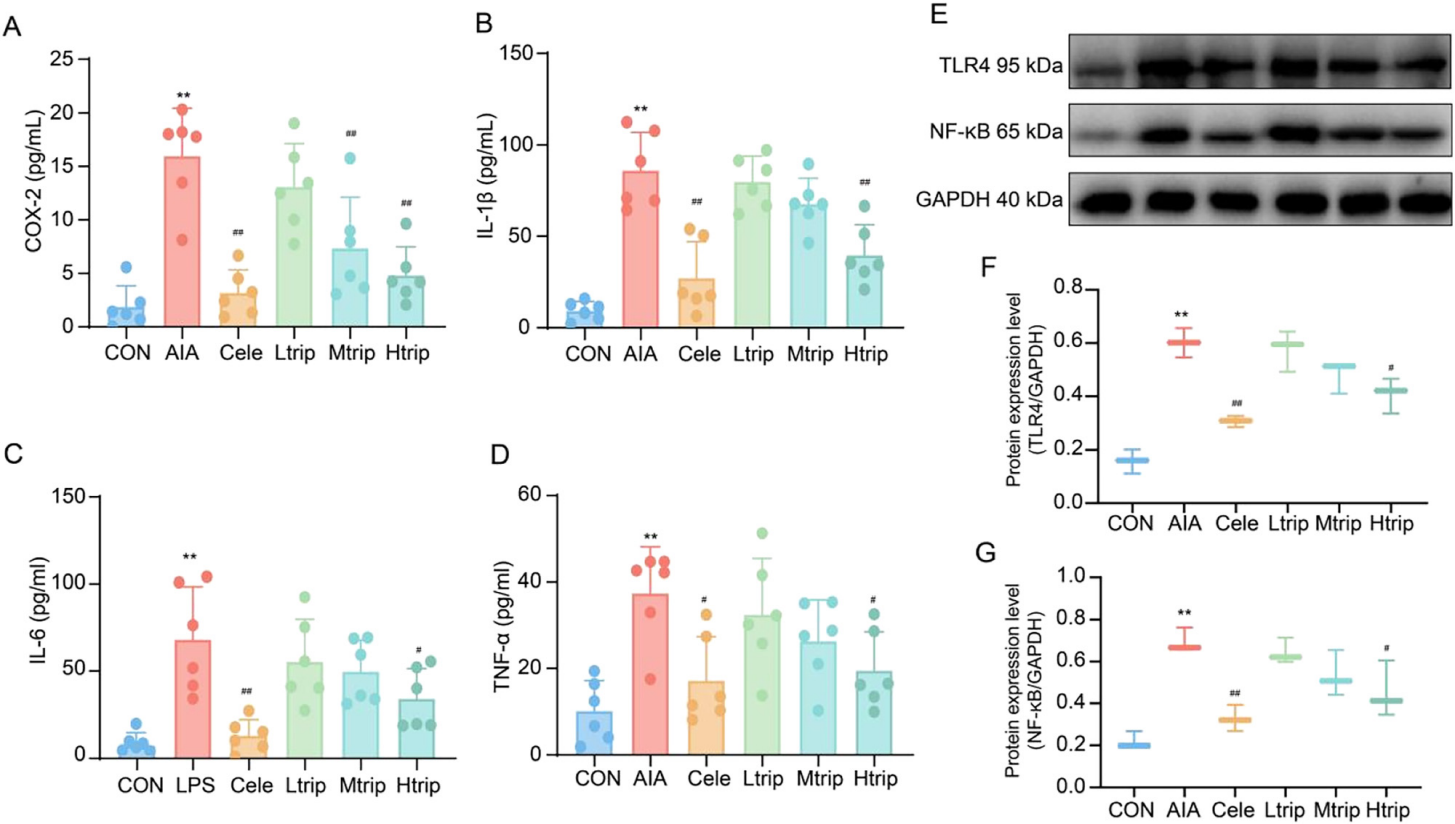
Effects of triptolide on the levels of inflammatory in the intestinal tissue of AIA mice. The levels of COX-2 (A), IL-1β (B), IL-6 (C) and TNF-α (D) in the supernatant were determined by ELISA. CON: Control group, AIA: adjuvant-induced arthritis group, Cele: Celecoxib group, Ltrip: low-dose triptolide group, Mtrip: medium-dose triptolide group, Htrip: high-dose triptolide group. Data are presented as the means ± SD (*n*=6 mice per group). ^**^ *p* < 0.01 vs. control group, ^#^ *p* < 0.05, ^##^ *p* < 0.01 vs. AIA model group. Effects of triptolide on TLR4 and NF-κB in AIA mice were measured by Western blotting analysis (E) Representative Western blot of target protein TLR4 and NF-κB expression. (F-G) Graph representing semi-quantitative analysis of the TLR4 and NF-κB proteins in each group, respectively. CON: Control group, AIA: adjuvant-induced arthritis group, Cele: Celecoxib group, Ltrip: low-dose triptolide group, Mtrip: medium-dose triptolide group, Htrip: high-dose triptolide group. Data are presented as the means ± SD from three independent experiments. ^**^ *p*  0.01 vs. control group, ^#^ *p* < 0.05, ^##^ *p* < 0.01 vs. AIA model group. One-way Analysis of Variance (One-way ANOVA) followed to Tukey’s Honestly Significant Difference (HSD) post-hoc test to evaluate overall group differences.

Cytokine levels were markedly elevated in AIA mice compared with controls, confirming a strong inflammatory response. Treatment with celecoxib or varying doses of triptolide effectively modulated cytokine production. Medium- and high-dose triptolide significantly reduced COX-2 expression, while only high-dose triptolide suppressed the levels of IL-1β, IL-6, and TNF-α Figure 5A–D. These findings align partially with the *in vitro* results, indicating that the therapeutic efficacy of triptolide in AIA mice is closely associated with a reduction in pro-inflammatory cytokine expression. The downregulation of these cytokines represents a key mechanism through which triptolide exerts its anti-inflammatory effects.

#### 3.2.4 Effect of triptolide on protein expression of TLR4/NF-κB pathway in synovial tissues of AIA mice.

*In vivo* analysis revealed that the NF-κB/TLR4 signaling pathway was markedly activated in the synovial tissues of AIA mice. Treatment with high-dose triptolide significantly inhibited the protein expression of TLR4 and NF-κB, whereas the medium dose produced no statistically significant changes Figure 5E–G. These findings suggest that triptolide exerts anti-inflammatory effects in AIA mice primarily through suppression of the TLR4/NF-κB signaling pathway.

#### 3.2.5 Effect of triptolide on protein and mRNA expression of ZO-1 and Occludin in AIA mice.

**Figure 6:**
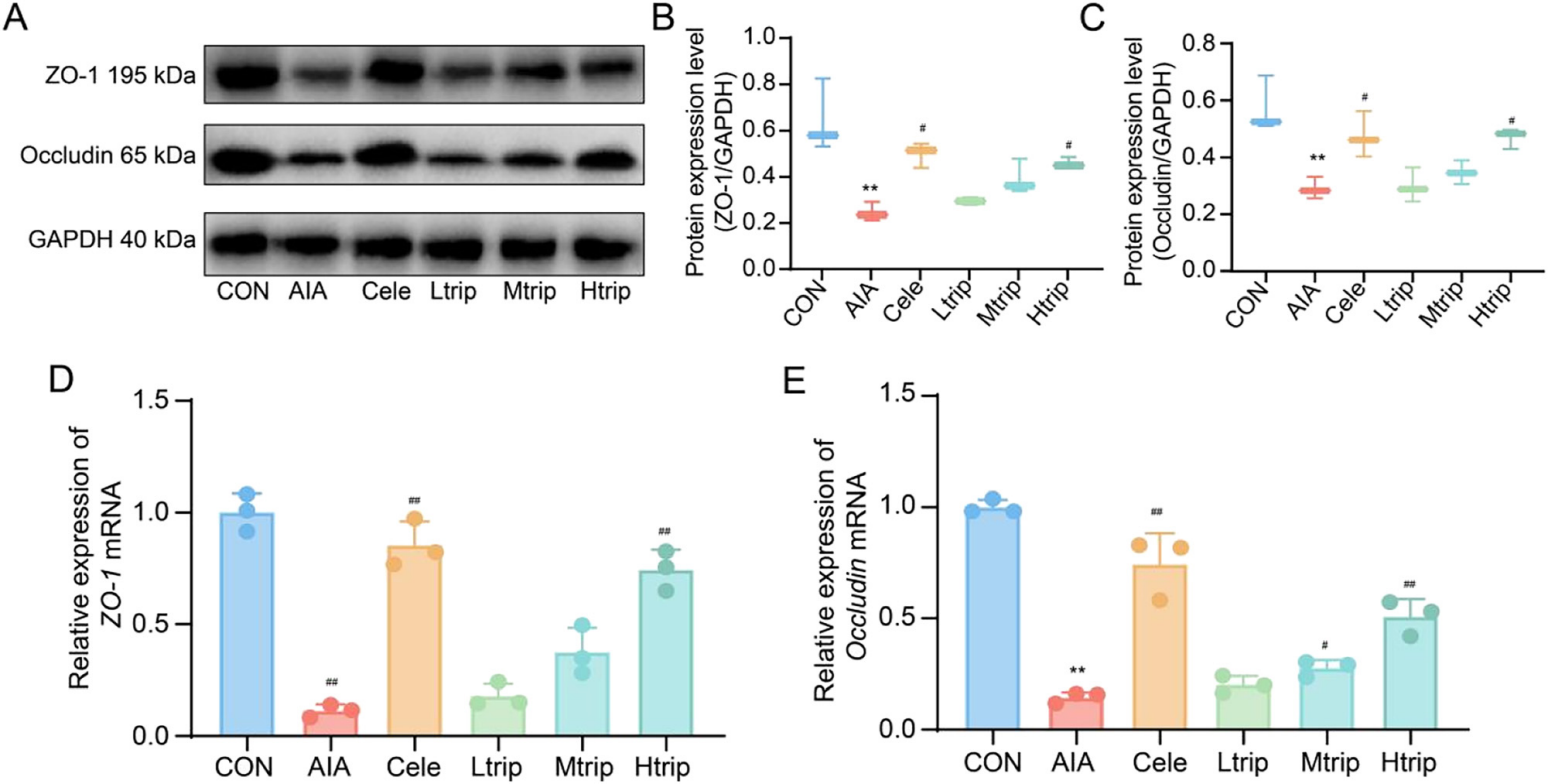
Effects of triptolide on ZO-1 and Occludin expression in intestinal tissues of AIA mice. (A) Representative Western blot of target protein ZO-1 and Occludin expression. (B-C) Graph representing semi-quantitative analysis of the TLR4 and NF-κB proteins in each group, respectively. (D-E) Relative mRNA expression levels of *ZO-1* and *Occludin* in intestinal tissues measured by reverse transcription-polymerase chain reaction (RT-PCR). Data are presented as the means ± SD from three independent experiments. CON: Control group, AIA: adjuvant-induced arthritis group, Cele: Celecoxib group, Ltrip: low-dose triptolide group, Mtrip: medium-dose triptolide group, Htrip: high-dose triptolide group. In mRNA expression analysis, data are presented as fold change compared to that of the control group. ^**^ *p *<* *0.01 vs. control group, ^#^ *p *<* *0.05, ^##^ *p *<* *0.01 vs. AIA model group. One-way Analysis of Variance (One-way ANOVA) followed to Tukey’s Honestly Significant Difference (HSD) post-hoc test to evaluate overall group differences.

Protein and mRNA expression levels of ZO-1 and Occludin are shown in Figure 6A–E. Both proteins were significantly downregulated in the AIA model group compared with controls (*p* < 0.05). Treatment with high-dose triptolide markedly increased the expression of ZO-1 and Occludin at both the protein and mRNA levels, while medium-dose triptolide produced no statistically significant changes (all *p* < 0.05). These findings indicate that Htrip enhances intestinal barrier integrity and alleviates inflammation by upregulating tight junction–associated proteins.

#### 3.2.6 Triptolide modulates the diversity and composition of intestinal microbiota in AIA mice

**Figure 7:**
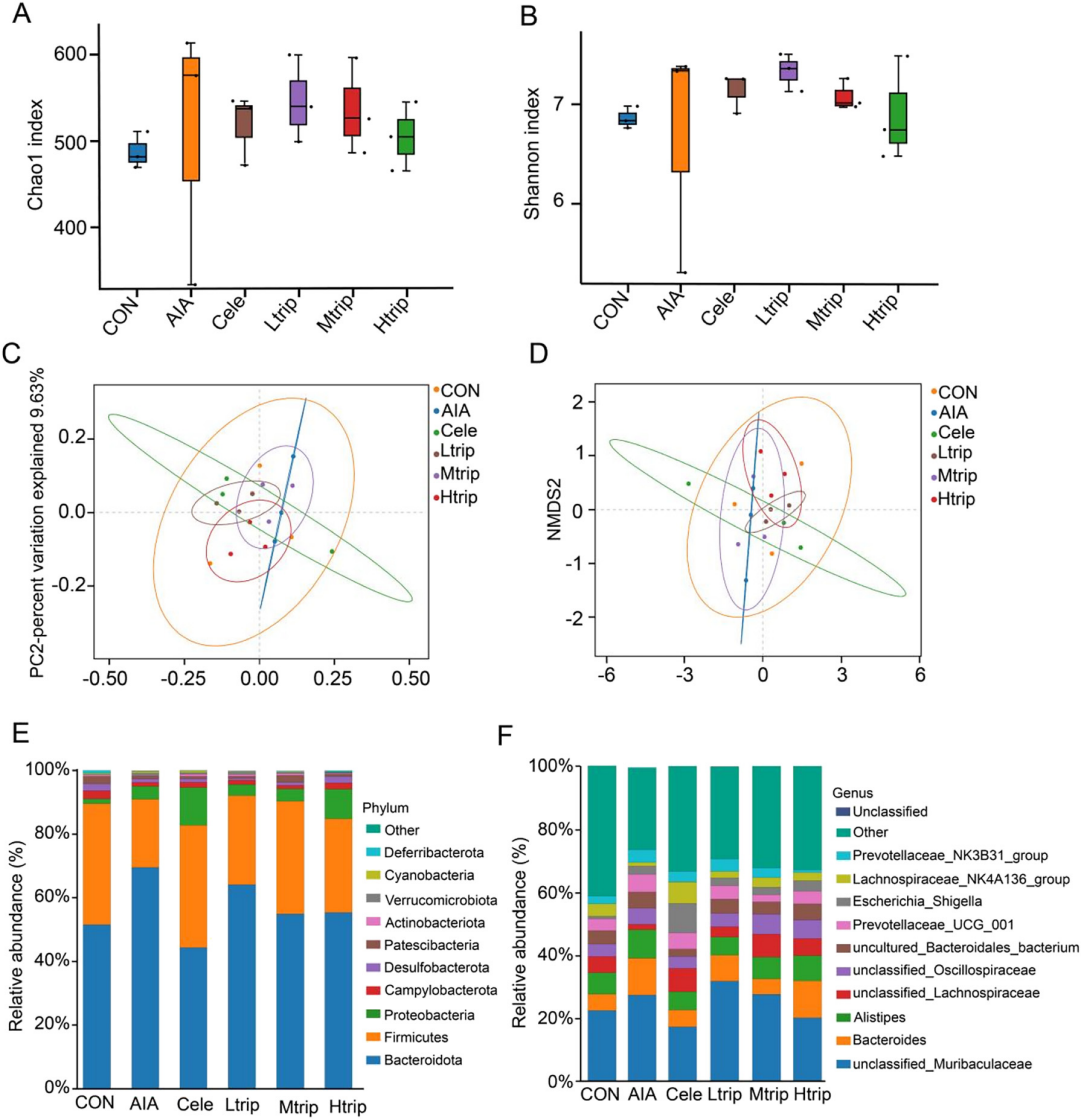
Triptolide modulates the diversity and composition of intestinal microbiota in AIA mice. (A) Chao1 index for Con, AIA, Htrip, Mtrip, Ltrip and Cele group; (B) Shannon index for Con, AIA, Htrip, Mtrip, Ltrip and Cele group. CON: Control group, AIA: adjuvant-induced arthritis group, Cele: Celecoxib group, Ltrip: low-dose triptolide group, Mtrip: medium-dose triptolide group, Htrip: high-dose triptolide group. Beta diversity at the phylum level in Con, AIA, Htrip, Mtrip, Ltrip and Cele group was assessed by (C) principal coordinate analysis (PCoA) and (D) non-metric multi-dimensional scaling (NMDS). CON: Control group, AIA: adjuvant-induced arthritis group, Cele: Celecoxib group, Ltrip: low-dose triptolide group, Mtrip: medium-dose triptolide group, Htrip: high-dose triptolide group. Relative abundance analyses of intestinal microbiota composition at the phylum (E) and gene (F) levels in Con, AIA, Htrip, Mtrip, Ltrip and Cele groups. CON: Control group, AIA: adjuvant-induced arthritis group, Cele: Celecoxib group, Ltrip: low-dose triptolide group, Mtrip: medium-dose triptolide group, Htrip: high-dose triptolide group.

Alpha diversity of the intestinal microbiota among six groups, including Control (Con), Adjuvant-Induced Arthritis (AIA), high-dose triptolide (Htrip), medium-dose triptolide (Mtrip), low-dose triptolide (Ltrip), and celecoxib (Cele), was evaluated using the Chao1 and Shannon indices. The Chao1 index reflects species richness within a sample, while the Shannon index represents overall diversity and evenness of microbial distribution. Both indices were significantly elevated in AIA mice compared with controls (*p* < 0.05), indicating increased microbial diversity during inflammation. Treatment with high- and medium-dose triptolide or celecoxib significantly reduced Chao1 and Shannon index values (*p* < 0.05) (Figure 7A-B), suggesting partial restoration of normal microbial balance. These findings demonstrate that triptolide and celecoxib modulated intestinal microbial diversity and improved community structure disrupted by AIA induction.

Beta-diversity analysis further revealed distinct clustering patterns among experimental groups. The microbial community structure of AIA mice differed markedly from that of the control group, as indicated by clear separation of data points in the PCA plot. Samples from the Htrip, Mtrip, and Cele group clustered closer to the control group, implying that medium- and high-dose triptolide, as well as celecoxib, effectively rebalanced the gut microbial composition altered by AIA (Figure 7C-D).

A taxonomic analysis was conducted to compare the intestinal microbial composition among the six groups. The relative abundance of *Bacteroidota* was markedly higher in the AIA group (70 %) than in the control group (52 %). Treatment with high- and medium-dose triptolide or celecoxib reduced the abundance of *Bacteroidota* to 55 %, 55 %, and 44 %, respectively. In contrast, the relative abundance of *Firmicutes* was significantly lower in the AIA group (22 %) compared with the control group (38 %). Administration of high- and medium-dose triptolide or celecoxib increased *Firmicutes* abundance to 30 %, 35 %, and 38 %, respectively. At the genus level, the abundance of *Bacteroides* was significantly elevated in the AIA group (12 %) compared with the control group (5 %). Treatment with high- and medium-dose triptolide or celecoxib reduced the abundance of *Bacteroides* to 5 %, 8 %, and 5 %, respectively (Figure 7E-F).

**Figure 8:**
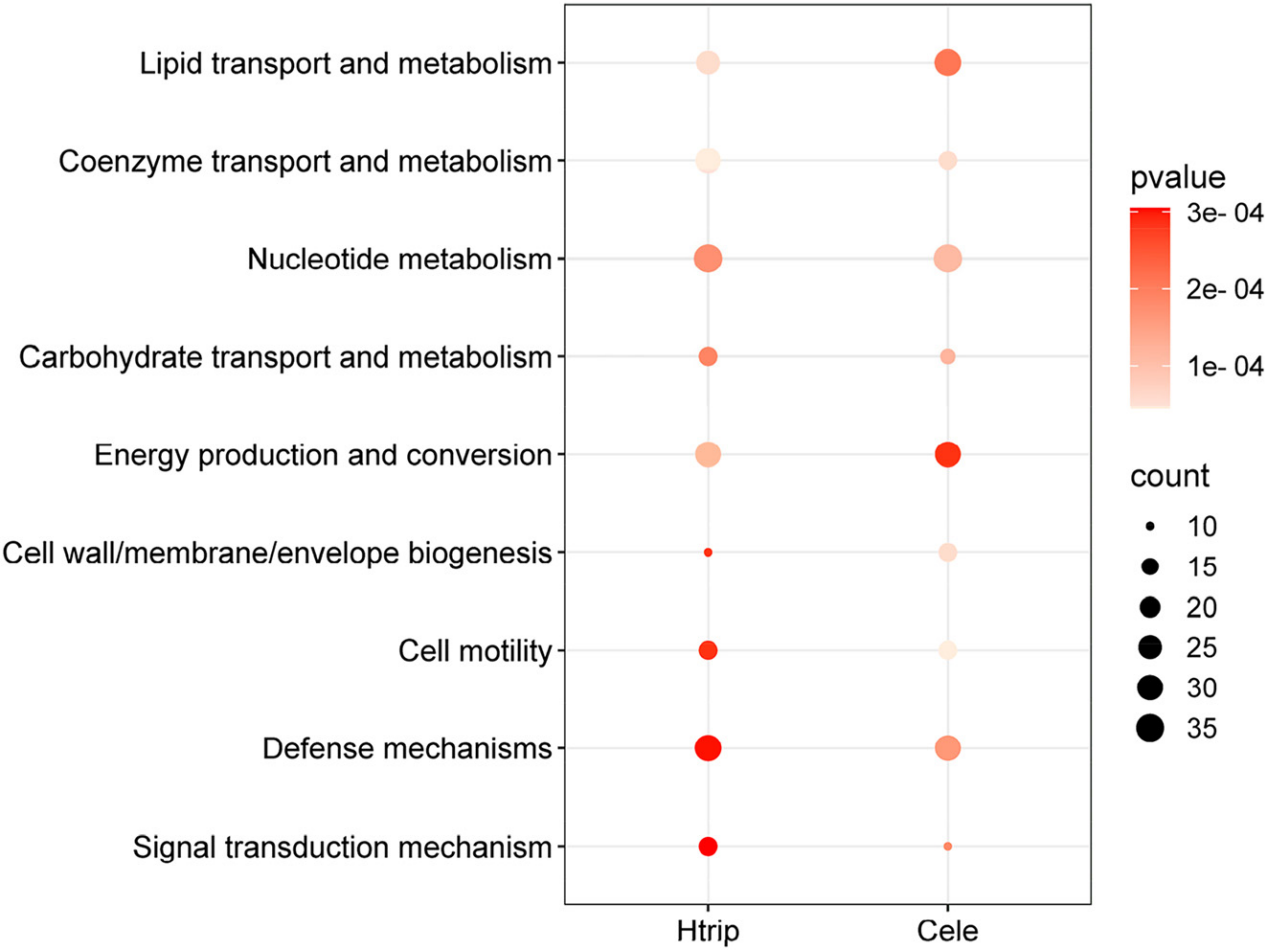
KEGG enrichment analysis of differentially expressed genes. Differentially expressed genes were analyzed for functional enrichment based on the Kyoto Encyclopedia of Genes and Genomes (KEGG) database. The pathways showing significant enrichment are presented, with corresponding genes associated with each pathway listed.

A Metastat analysis was conducted to identify bacterial taxa that were differentially abundant among the six groups. At the phylum level, *Actinobacteriota* and *Patescibacteria* were significantly upregulated, whereas *Proteobacteria* showed a marked reduction. The *Bacteroidota* taxa were enriched in the celecoxib-treated group, while *Acidobacteriota*, *Firmicutes*, and *Bacteroidota* were enriched in the medium-dose triptolide group. Additionally, several bacterial taxons, including *Lachnospiraceae_UCG_010*, *Anaerofustis*, *Lactobacillus*, *unclassified_Erysipelotri-chaceae*, *Candidatus_Arthromitus*, *A2, Prevotellaceae_UCG_001*, *Achromobacter*, *Sporosarcina*, and *[Eubacterium]_nodatum_group* were significantly altered in the AIA group, while others (*uncultured_Bacteroidales_bacterium*, *Turicibacter, unclassified_Muribaculaceae, Achromobacter, Romboutsia, Rikenella, Odoribacter,* and *Anaeroplasma*) were enriched in the Cele intervention group, and *Gordonibacter, A2*, *[Eubacterium]_nodatum_group*, *[Eubacterium]_xylanophilum_group*, *Prevotell-aceae_UCG_001*, *Achromobacter*, *Parvibacter*, *Lachnospiraceae_FCS020_group*, *ASF356*, *unclassified_Lachnospiraceae*, and *Anaerotruncus* taxons, were enriched in the medium-dose triptolide intervention group (Supplementary Tables 1–6). Functional enrichment analysis using the KEGG database demonstrated that the differentially expressed genes (DEGs) identified from these taxa participated in multiple biological processes and signaling pathways related to immune regulation, metabolism, and inflammation. The specific genes associated with these enriched pathways are listed in Figure 8.

## 4 Discussion

The interplay between gut microbiota dysregulation, intestinal barrier dysfunction, and systemic inflammation is increasingly recognized as a central driver of RA pathogenesis. The present study demonstrates that triptolide alleviates RA in mice by reshaping gut microbial composition, repairing intestinal barrier integrity, and suppressing the TLR4/NF-κB signaling pathway. These findings extend beyond descriptive observations to reveal mechanistic links between microbial modulation and immune regulation, offering new insights into triptolide’s therapeutic potential and the gut-joint axis in RA.

Triptolide, a diterpenoid from *Tripterygium wilfordii*, has long been studied for its anti-inflammatory and immunosuppressive properties in RA. Lu et al. found triptolide can provide protective effects in the collagen-induced arthriti and bleomycin (BLM)-induced pulmonary fibrosis model by reducing arthritis and pulmonary fibrosis through the inhibition of insulin-like growth factor (IGF1)-mediated epithelial-mesenchymal transitio (EMT) [16]. Additionally, by acting on the RhoA/Rho-associated kinase signaling pathway, triptolide regulates actin cytoskeleton remodeling, thereby inhibiting the motility of rheumatoid arthritisfibroblast-like synoviocytes (RA-FLS). Through comprehensive review, Wang et al. reported that triptolide can act on monocytes/macrophages, dendritic cells, T cells, fibroblast-like synoviocytes, and osteoclasts through regulating multiple key signaling pathways such as NF-κB, JAK/STAT, and MAPK [20]. Although these progresses, a crucial issue has been overlooked, that is, as a “hidden immune organ”, gut microbiota paly a crucial role in immune and inflammatory diseases. To uncover this issue, we analyzed the anti-RA impact and mechanisms of triptolide from the gut microbiota perspective, contributing additional insights to previous research.

Generally, the gut microbiota interacts with the host immune system via direct contact, metabolite signaling, and antigen presentation, forming a tightly regulated network critical for immune function. Firstly, the gut microbiota strengthens the intestinal mechanical barrier by promoting the expression of tight junction proteins (e.g., ZO-1, Occludin) and mucus production (via goblet cells). The intestinal barrier serves as a critical defense system that separates luminal contents from host tissues and prevents the invasion of pathogenic antigens. Tight junctions between adjacent intestinal epithelial cells, primarily formed by ZO-1 and Occludin, create a tightly linked complex that anchors to the cytoskeleton, effectively sealing intercellular spaces [21]. In colitis mouse models, the expression of ZO-1 and Occludin is significantly downregulated [22], and a similar reduction has been observed in the intestinal tissues of patients with RA [23]. The present study examined the intestinal mucosa of AIA mice to determine how triptolide influences intestinal permeability. Treatment with triptolide markedly increased ileal length, enhanced nutrient absorption, and improved intestinal integrity by repairing mucosal damage, as evidenced by Western blot analysis. The upregulation of ZO-1 and Occludin expression reduced intestinal permeability, strengthened the mechanical barrier, and limited the activation of autoimmune responses triggered by pathogenic microorganisms. Additionally, commensal bacteria release microbe-associated molecular patterns (MAMPs), such as lipopolysaccharide (LPS, from Gram-negative bacteria) and peptidoglycan, which bind to PRRs (e.g., TLR4, NOD2) on immune cells. This “tonic stimulation” primes innate immunity without triggering inflammation, enabling rapid responses to pathogens. The activation of TLR4 in RA mice suggests the disruption of gut microbiota in RA mice and upregulation of pathogenic bacteria, which further leading to the release of LPS and TLR4 activation. These results suggest that triptolide ameliorates RA, possibly via reversing dysbiosis of the gut microbiota.

As for the changes of gut microbial composition, we found triptolide decreased *Bacteroidota* but increased *Firmicutes*. Commonly, the Firmicutes/Bacteroidota (F/B) ratio is often cited as a marker of gut dysbiosis, but its role in RA remains controversial. Gabriel et al. reported that patients with RA exhibit altered intestinal compositions of *Firmicutes* and *Bacteroidetes* [7]. The changes in the Firmicutes/Bacteroidetes ratio can impair intestinal barrier integrity and immune regulation, allowing conditional pathogenic bacteria to invade, enhancing immune responses to exogenous antigens, and activating the TLR4-mediated NF-κB signaling pathway, thereby amplifying inflammation [24]. Huang et al. reported that *Qingluo Tongbi* Decoction reduces TLR4 expression in the synovial tissue of AIA mice by modulating the relative abundance ratio of *Firmicutes* and *Bacteroidetes* [25]. Consistent with these findings, triptolide was shown to restore intestinal microbial balance by increasing *Firmicutes*, decreasing *Bacteriodota*, and suppressing activation of the TLR4/NF-κB pathway. Mechanistically, *Firmicutes* harbor several butyrate-producing bacteria that contribute to intestinal health. Butyrate enhances intestinal barrier integrity, stabilizes the intestinal microenvironment, and modulates both inflammation and immune function [26]. Additionally, Proteobacteria, possessing a higher number of LPS-producing bacteria, was increased in RA mice, indicating higher LPS availability in circulation may drive excessive activation of the TLR4-NF-κB signaling axis, initiating a destructive cascade of pro-inflammatory responses. Triptolide may improve RA by balancing the microbiota, reducing the production of LPS, and thereby inhibiting the TLR4/NF-κB signaling pathway.

Several limitations should be acknowledged. The current study relied solely on animal and cellular experiments, without clinical validation. Further investigations are necessary to identify microbiological targets for triptolide intervention in RA through clinical trials. Moreover, the analysis was confined to 16S rRNA sequencing, whereas metagenomic approaches could provide deeper insights into the effects of triptolide on intestinal microbiota in RA. Finally, the speculation of “gut microbiota–intestinal barrier–TLR4/NF-κB axis” in triptolide’s anti-RA effect should be verified in the future comprehensive study.

## 5 Conclusions

**Figure 9:**
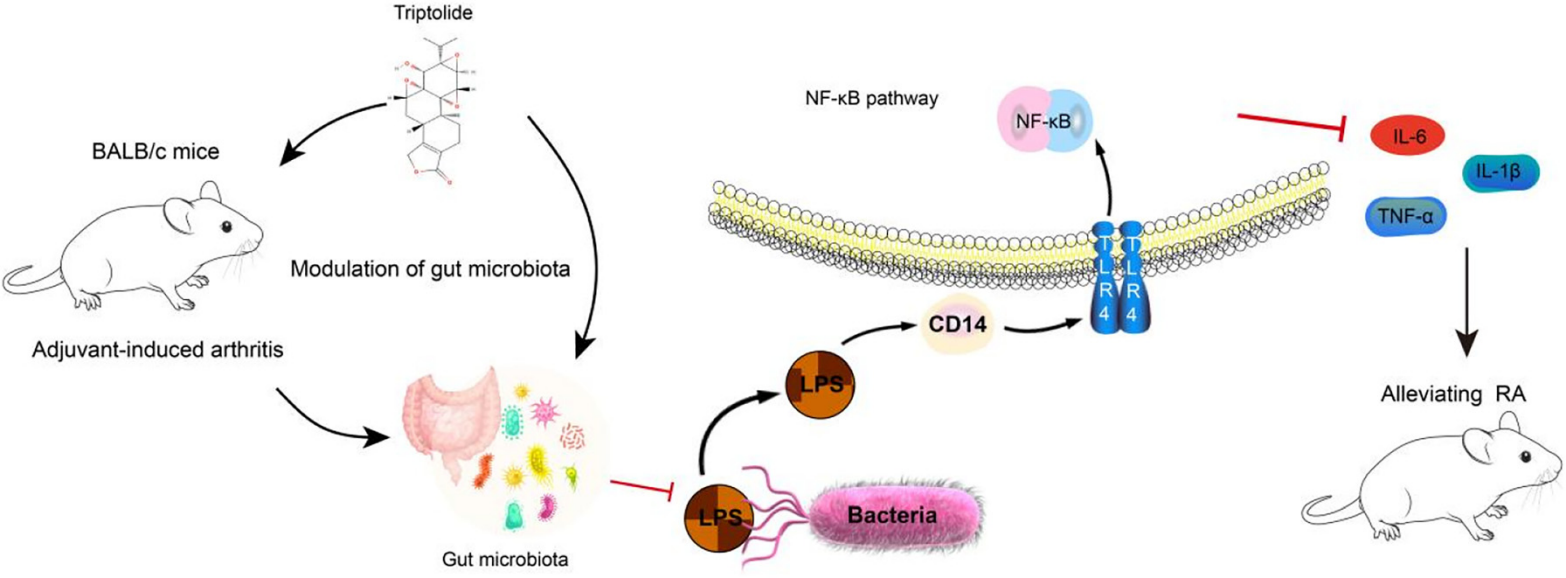
Triptolide alleviates RA through modulation of “microbiota-intestinal barrier-TLR4/NF-κB Axis”.

Overall, triptolide showed a strong capacity to alleviate inflammatory responses in AIA mice and promote intestinal barrier repair by upregulating ZO-1 and Occludin expression. The therapeutic mechanism appears to be mediated through modulation of the intestinal microbiota and suppression of the TLR4/NF-κB signaling pathway (Figure 9), ultimately contributing to the attenuation of inflammation and restoration of immune homeostasis.

## Supplementary Material

Supplementary Material
