# Supplementary material for: Triptolide alleviates rheumatoid arthritis via modulating gut microbiota – intestinal barrier – TLR4/NF-κB axis
Source: Open Life Sci. 2026 Jun 8;21(1):20251290. doi: 10.1515/biol-2025-1290 (PMC13241201; doi:10.1515/biol-2025-1290)
Supplement: Supplementary file 1 — Supplementary Material [file j_biol-2025-1290_suppl_001.docx]

**Supplementary Table S1:** Metastat analysis between the AIA group and the control group at the phylum level.

| **Phylum** | **Group** | **Mean (se)** | ***P*-value** |
| --- | --- | --- | --- |
| Actinobacteriota | AIA | 0.00202 (0.001617 - 0.002425) | 0.00671** |
|  | sham | 0.00456 (0.003905 - 0.005215) |  |
| Proteobacteria | AIA | 0.0407 (0.0352 - 0.0462) | 0.0139* |
|  | sham | 0.0156 (0.00861 - 0.02259) |  |
| Patescibacteria | AIA | 0.00909 (0.00759 - 0.01059) | 0.0343* |
|  | sham | 0.0226 (0.01667 - 0.02853) |  |
| Methyloirregularia | AIA | 0 (0 - 0) | 0.066 |
|  | sham | 1.96e-05 (9.79e-06 - 2.941e-05) |  |
| Firmicutes | AIA | 0.215 (0.20957 - 0.22043) | 0.207 |
|  | sham | 0.38 (0.275 - 0.485) |  |
| Bacteroidota | AIA | 0.696 (0.69428 - 0.69772) | 0.229 |
|  | sham | 0.516 (0.389 - 0.643) |  |
| Campylobacterota | AIA | 0.012 (0.00995 - 0.01405) | 0.25 |
|  | sham | 0.0257 (0.015 - 0.0364) |  |
| Desulfobacterota | AIA | 0.011 (0.010175 - 0.011825) | 0.285 |
|  | sham | 0.0223 (0.0123 - 0.0323) |  |
| Verrucomicrobiota | AIA | 0.00729 (0.00127 - 0.01331) | 0.315 |
|  | sham | 0.00396 (0.000391 - 0.001531) |  |
| Deferribacterota | AIA | 0.00144 (0.001224 - 0.001656) | 0.349 |
|  | sham | 0.00821 (0.00161 - 0.01481) |  |
| Acidobacteriota | AIA | 9.81e-06 (0 - 1.962e-05) | 0.362 |
|  | sham | 2.94e-05 (1.24e-05 - 4.64e-05) |  |
| Calditrichota | AIA | 9.81e-06 (0 - 1.962e-05) | 0.5 |
|  | sham | 0 (0 - 0) |  |
| Dadabacteria | AIA | 9.81e-06 (0 - 1.962e-05) | 0.5 |
|  | sham | 0 (0 - 0) |  |
| unclassified_Bacteria | AIA | 4.9e-05 (1.92e-05 - 7.88e-05) | 0.633 |
|  | sham | 3.43e-05 (2.13e-05 - 4.73e-05) |  |
| Cyanobacteria | AIA | 0.00489 (0.00224 - 0.00754) | 0.782 |
|  | sham | 0.00415 (0.00135 - 0.00695) |  |

**Supplementary Table S2:** Metastat analysis between the AIA group and Cele group at the phylum level.

| **Phylum** | **Group** | **Mean (se)** | ***P*-value** |
| --- | --- | --- | --- |
| Bacteroidota | AIA | 0.696 (0.69428 - 0.69772) | 0.00815** |
|  | Cele | 0.444 (0.3576 - 0.5304) |  |
| Firmicutes | AIA | 0.215 (0.20967 - 0.22043) | 0.391 |
|  | Cele | 0.384 (0.205 - 0.563) |  |
| Actinobacteriota | AIA | 0.00202 (0.001617 - 0.002423) | 0.423 |
|  | Cele | 0.00677 (0.00144 - 0.0121) |  |
| Calditrichota | AIA | 9.81e-06 (0-1.962e-05) | 0.5 |
|  | Cele | 0 (0 - 0) |  |
| Dadabacteria | AIA | 9.81e-06 (0 - 1.962e-05) | 0.5 |
|  | Cele | 0 (0 - 0) |  |
| Proteobacteria | AIA | 0.0407 (0.0352 - 0.0462) | 0.546 |
|  | Cele | 0.12 (0.0030 - 0.237) |  |
| Campylobacterota | AIA | 0.012 (0.00995 - 0.01405) | 0.585 |
|  | Cele | 0.0155 (0.01035 - 0.02065) |  |
| unclassified_Bacteria | AIA | 4.9e-05 (1.92e-05 - 7.88e-05) | 0.616 |
|  | Cele | 9.81e-05 (1.43e-05 - 0.0001819) |  |
| Verrucomicrobiota | AIA | 0.00729 (0.00127 - 0.01331) | 0.685 |
|  | Cele | 0.00493 (0.00036 - 0.0095) |  |
| Patescibacteria | AIA | 0.00909 (0.00759 - 0.01059) | 0.717 |
|  | Cele | 0.00791 (0.00413- 0.01169) |  |
| Acidobacteriota | AIA | 9.81e-06 (0 - 1.962e-05) | 0.725 |
|  | Cele | 1.47e-05 (0 - 2.94e-05) |  |
| Deferribacterota | AIA | 0.00144 (0.001224 - 0.001656) | 0.839 |
|  | Cele | 0.00137 (0.000918 - 0.001822) |  |
| Cyanobacteria | AIA | 0.00489 (0.00224 - 0.00754) | 0.884 |
|  | Cele | 0.00455 (0.00085 -0.00825) |  |
| Desulfobacterota | AIA | 0.011 (0.010175 - 0.011825) | 0.9 |
|  | Cele | 0.0112 (0.0076 - 0.0148) |  |
| BDellovibrionota | AIA | 0 (0 - 0) | 1 |
|  | Cele | 4.9e-06 (0 - 9.8e-06) |  |

**Supplementary Table S3:** Metastat analysis between the AIA group and Mtrip group at the phylum level.

| **Phylum** | **Group** | **Mean (se)** | ***P*-value** |
| --- | --- | --- | --- |
| Acidobacteriota | AIA | 9.81e-06 (0 - 1962e-05) | 0.0135* |
|  | Mtrip | 0.000127 (9.19e-05 -0.0001624) |  |
| Firmicutes | AIA | 0.215 (0.20967 - 0.22043) | 0.0261* |
|  | Mtrip | 0.354 (0.2964 - 0.4116) |  |
| Bacteroidota | AIA | 0.696 (0.69428 - 0.69772) | 0.0389* |
|  | Mtrip | 0.552 (0.4859 - 0.6181) |  |
| Gemmatimonadota | AIA | 0 (0 - 0) | 0.102 |
|  | Mtrip | 2.94e-05 (1.24e-05 - 4.64e-05) |  |
| Actinobacteriota | AIA | 0.00202 (0.001617 - 0.002423) | 0.134 |
|  | Mtrip | 0.00652 (0.00363 - 0.00941) |  |
| unclassified_Bacteria | AIA | 4.9e-05 (1.92e-05 - 7.88e-05) | 0.141 |
|  | Mtrip | 4.9e-06 (0 - 9.8e-06) |  |
| Patescibacteria | AIA | 0.00909 (0.00759 - 0.01059) | 0.169 |
|  | Mtrip | 0.0224 (0.0133 - 0.0315) |  |
| Desulfobacterota | AIA | 0.011 (0.010175- 0.011825) | 0.25 |
|  | Mtrip | 0.00871 (0.00714 - 0.01028) |  |
| Cyanobacteria | AIA | 0.00489 (0.00224 - 0.00754) | 0.469 |
|  | Mtrip | 0.0026(0.0025305 - 0.0026695) |  |
| Verrucomicrobiota | AIA | 0.00729 (0.00127 - 0.01331) | 0.487 |
|  | Mtrip | 0.00214 (0.001343 - 0.002937) |  |
| Dadabacteria | AIA | 9.81e-06 (0 - 1962e-05) | 0.5 |
|  | Mtrip | 0 (0 - 0) |  |
| Calditrichota | AIA | 9.81e-06 (0 - 1962e-05) | 0.747 |
|  | Mtrip | 1.47e-05 (6.21e-06 - 2.319e-05) |  |
| Deferribacterota | AIA | 0.00144 (0.001224 - 0.001656) | 0.787 |
|  | Mtrip | 0.00175 (0.00096 - 0.00254) |  |
| Campylobacterota | AIA | 0.012 (0.00995 - 0.01405) | 0.881 |
|  | Mtrip | 0.0109 (0.00546 - 0.01634) |  |
| Proteobacteria | AIA | 0.0407 (0.0352 - 0.0462) | 0.913 |
|  | Mtrip | 0.0391 (0.006 - 0.0722) |  |

**Supplementary Table S4:** Metastat analysis between the AIA group and Con group at the genus level.

| **Genus** | **Group** | **Mean (se)** | ***P*-value** |
| --- | --- | --- | --- |
| *Lachnospiraceae_UCG_010* | AIA | 0 (0 - 0) | <0.001*** |
|  | sham | 4.41e-05 (4.41e-05 - 4.41e-05) |  |
| *Anaerofustis* | AIA | 5.39e-05 (4.409e-05 - 6.371e-05) | 0.0085** |
|  | sham | 4.9e-06 (0 - 9.8e-06) |  |
| *Lactobacillus* | AIA | 0.00891 (0.00295 - 0.01487) | 0.0112* |
|  | sham | 0.0359 (0.03226 - 0.03954) |  |
| *unclassified_Erysipelotrichaceae* | AIA | 0.00225 (0.002107 - 0.002393) | 0.0119* |
|  | sham | 0.0015 (0.001365 - 0.001635) |  |
| *Candidatus_Arthromitus* | AIA | 1.47e-05 (6.216-06 - 2.319e-05) | 0.0212* |
|  | sham | 0.000157 (0.0001102 - 0.0002038) |  |
| *A2* | AIA | 0 (0 - 0) | 0.0219* |
|  | sham | 0.000127 (8.43e-05 - 0.0001697) |  |
| *Prevotellaceae_UCG_001* | AIA | 0.0558 (0.05032 - 0.06128) | 0.0284* |
|  | sham | 0.0358 (0.03139 - 0.04021) |  |
| *Achromobacter* | AIA | 0.000142 (0.0001019 - 0.0001821) | 0.0298* |
|  | sham | 2.45e-05 (1.15e-05 - 3.75e-05) |  |
| *Sporosarcina* | AIA | 0.00025 (0.000136 - 0.000364) | 0.0318* |
|  | sham | 0.0032 (0.00211 - 0.00429) |  |
| *[Eubacterium]_nodatum_group* | AIA | 0.00029 (0.0002283 - 0.0003317) | 0.0338* |
|  | sham | 0.000637 (0.00051 - 0.000764) |  |

**Supplementary Table S5:** Metastat analysis between the AIA group and Cele group at the genus level.

| **Genus** | **Group** | **Mean (se)** | ***P*-value** |
| --- | --- | --- | --- |
| *uncultured_Bacteroidales_bacterium* | AIA | 0.0516 (0.04978 - 0.05342) | <0.001*** |
|  | Cele | 0.0248 (0.02074 - 0.02886) |  |
| *Turicibacter* | AIA | 0.000289 (0.0002407 - 0.0003373) | 0.00137** |
|  | Cele | 0 (0 - 0) |  |
| *unclassified_Muribaculaceae* | AIA | 0.277 (0.2648 - 0.2892) | 0.00366** |
|  | Cele | 0.171 (0.1555 - 0.1865) |  |
| *Achromobacter* | AIA | 0.000142 (0.0001019 - 0.0001821) | 0.0129* |
|  | Cele | 0 (0 - 0) |  |
| *Romboutsia* | AIA | 5.39e-05 (3.62e-05 - 7.16e-05) | 0.0199* |
|  | Cele | 0 (0 - 0) |  |
| *Rikenella* | AIA | 0.00618 (0.00493 - 0.00743) | 0.0221* |
|  | Cele | 0.00216 (0.001718 - 0.002602) |  |
| *Odoribacter* | AIA | 0.0121 (0.01054 - 0.01366) | 0.031* |
|  | Cele | 0.00499 (0.00267 - 0.00731) |  |
| *Clostridium_sensu_stricto_1* | AIA | 0.000333 (2e-04 - 0.000466) | 0.0362* |
|  | Cele | 4.9e-06 (0 - 9.8e-06) |  |
| *Anaeroplasma* | AIA | 0.000333 (0.000203 - 0.000463) | 0.048* |
|  | Cele | 2.94e-05 (6.9e-06 - 5.19e-05) |  |

**Supplementary Table S6:** Metastat analysis between the AIA group and Mtrip group at the genus level.

| **Genus** | **Group** | **Mean (se)** | ***P*-value** |
| --- | --- | --- | --- |
| *Gordonibacter* | AIA | 4.9e-05 (2.45e-05 - 7.35e-05) | <0.001*** |
|  | Mtrip | 0.000456 (0.0004413 - 0.0004707) |  |
| *A2* | AIA | 0 (0 - 0) | 0.00292** |
|  | Mtrip | 0.000485 (0.0004262 - 0.0005438) |  |
| *[Eubacterium]_nodatum_group* | AIA | 0.00028 (0.0002283- 0.0003317) | 0.0036** |
|  | Mtrip | 0.00076 (0.0007047 - 0.0008153) |  |
| *[Eubacterium]_xylanophilum_group* | AIA | 0.000794 (0.000382 - 0.001206) | 0.00492** |
|  | Mtrip | 0.0104 (0.00875 - 0.01205) |  |
| *Prevotellaceae_UCG_001* | AIA | 0.0558 (0.05032 - 0.06128) | 0.0126* |
|  | Mtrip | 0.0231 (0.01525 - 0.03095) |  |
| *Achromobacter* | AIA | 0.000142 (0.0001019 - 0.0001821) | 0.0141* |
|  | Mtrip | 4.9e-06 (0 - 9.8e-06) |  |
| *Parvibacter* | AIA | 5.39e-05 (2.41e-05 - 8.37e-05) | 0.0168* |
|  | Mtrip | 0.000167 (0.0001456 - 0.0001884) |  |
| *Lachnospiraceae_FCS020_group* | AIA | 0.000226 (0.0001324 - 0.0003196) | 0.0174* |
|  | Mtrip | 0.000701 (0.000577 - 0.000825) |  |
| *ASF356* | AIA | 0.00024 (0.000125 - 0.000355) | 0.0203* |
|  | Mtrip | 0.00168 (0.001214 - 0.002146) |  |
| *unclassified_Lachnospiraceae* | AIA | 0.0176 (0.01478 - 0.02042) | 0.0261* |
|  | Mtrip | 0.0731 (0.0532 - 0.093) |  |
| *Anaerotruncus* | AIA | 0.000167 (9.48e-05 - 0.0002392) | 0.0296* |
|  | Mtrip | 0.000912 (0.000642 - 0.001182) |  |
| *unclassified_Vicinamibacteraceae* | AIA | 0 (0 - 0) | 0.0378* |
|  | Mtrip | 2.45e-05 (1.469e-05 - 3.431e-05) |  |
| *unclassified_Peptococcaceae* | AIA | 0.000402 (0.000296 - 0.000508) | 0.047* |
|  | Mtrip | 0.00103 (0.000781 - 0.001279) |  |
| *unclassified_Eggerthellaceae* | AIA | 0.000162 (5.4e-05- 0.00027) | 0.0489* |
|  | Mtrip | 0.000755 (0.000523 - 0.000987) |  |
